# A Fucosylated Glycopeptide as a Candidate Biomarker for Early Diagnosis of NASH Hepatocellular Carcinoma Using a Stepped HCD Method and PRM Evaluation

**DOI:** 10.3389/fonc.2022.818001

**Published:** 2022-03-17

**Authors:** Yu Lin, Jie Zhang, Ana Arroyo, Amit G. Singal, Neehar D. Parikh, David M. Lubman

**Affiliations:** ^1^ Department of Surgery, University of Michigan Medical Center, Ann Arbor, MI, United States; ^2^ Department of Internal Medicine, University of Texas Southwestern Medical Center, Dallas, TX, United States; ^3^ Department of Internal Medicine, University of Michigan Medical Center, Ann Arbor, MI, United States

**Keywords:** fucosylated glycopeptides, ceruloplasmin, stepped-HCD MS/MS, NASH HCC, biomarkers

## Abstract

Aberrant specific N-glycosylation, especially the increase in fucosylation on specific peptide sites of serum proteins have been investigated as potential markers for diagnosis of nonalcoholic steatohepatitis (NASH)-related HCC. We have combined a workflow involving broad scale marker discovery in serum followed by targeted marker evaluation of these fucosylated glycopeptides. This workflow involved an LC-Stepped HCD-DDA-MS/MS method coupled with offline peptide fractionation for large-scale identification of *N*-glycopeptides directly from pooled serum samples (each n=10) as well as differential determination of N-glycosylation changes between disease states. We then evaluated the fucosylation level of the glycoprotein ceruloplasmin among 62 patient samples (35 cirrhosis, 27 early-stage NASH HCC) by LC-Stepped HCD-PRM-MS/MS to quantitatively analyze 18 targeted glycopeptides. Of these targets, we found the ratio of fucosylation of a tri-antennary glycopeptide from site N762, involving N762_ HexNAc(5)Hex(6)Fuc(2)NeuAc(3) (P=0.0486), increased significantly from cirrhosis to early HCC. This fucosylation ratio of a tri-antennary glycopeptide in CERU could be a potential biomarker for further validation in a larger sample set and could be a promising candidate for early detection of NASH HCC.

**Graphical Abstract:**
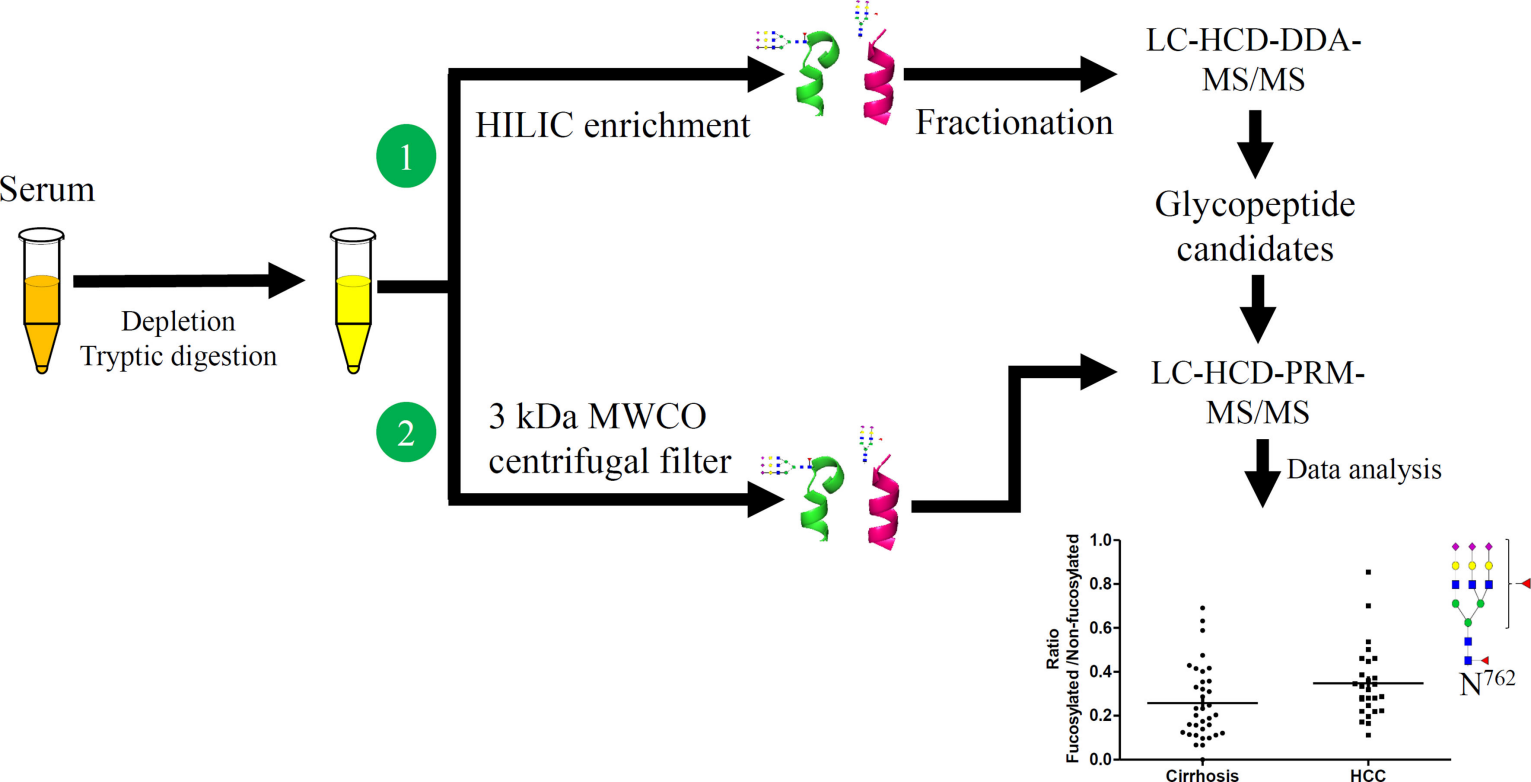


## Introduction

Hepatocellular carcinoma (HCC) is the 3^rd^ most common cause of cancer-related death worldwide (1). Most HCC cases develop in the setting of underlying cirrhosis. Changes in glycosylation in serum proteins are often associated with HCC development where glycosylation can act as a critical regulatory mechanism. Thus, the changes of these glycopeptides may be potential markers for HCC diagnosis and early detection. Many serum proteins are synthesized and secreted from the liver, thus, the aberrant glycosylation serum proteins are able to serve as potential biomarkers of liver disease progression (2). Serum markers including alpha fetoprotein (AFP) and AFP-L3, a core-fucosylated form of AFP, have been used in clinical settings for early detection monitoring. However, these markers still have inadequate performance characteristics for early detection (3, 4). To date, there are data available on several serum proteins as potential biomarkers for HCC detection; however, most existing markers require further validation prior to use in routine clinical practice (5). Hence, new diagnostic strategies and biomarkers are urgently needed for early HCC detection.

Recent advances in mass spectrometry (MS) combined with glycopeptide enrichment methods provide an opportunity for glycopeptide identification and quantitation. HCD (higher-energy collision dissociation) MS fragmentation techniques can identify intact glycopeptides, which allows fragment ion detection with great accuracy at low m/z values with only 100 ms fragmentation cycles. This capability provides a means to quickly scan for glycan diagnostic oxonium ions and to analyze more complex glycopeptides (6), where a thousand N-glycosylation sites have been identified from human liver tissue and serum. Some glycopeptide enrichment methods, such as lectins (7–9), solid-phase extraction (10), and hydrophilic interaction liquid chromatography (HILIC) (11–13), have been used to profile the N-glycopeptides (14).

The fucosylation of glycopeptides can be used as potential markers for early detection of various cancers (15). The ratio of core-fucosylated glycopeptides from ceruloplasmin (CERU), a serum glycoprotein, are increased in patients with alcohol-related, hepatitis B (HBV) and hepatitis C (HCV) (16). Another marker candidate for HCC early detection is the structure–specific fucosylation of alpha-1-antitrypsin, another serum glycoprotein (17, 18). Multi-fucosylated alpha-1-acid glycoprotein, especially the outer arm fucosylated glycan form, has undergone early validation as a novel marker for HBV and HCV HCC (8).

Serum haptoglobin (Hp), containing four glycosites, is an abundant glycoprotein secreted into the blood primarily by the liver, where its major function is modulating renal iron loading, and to prevent kidney damage by released iron. Hp has been reported as a reporter protein for aberrant glycosylations in patients with HCC (19). Hp protein glycosylation is characterized by both outer arm multi-fucosylation and core-fucosylated glycopeptides (20–22). These results indicated that the fucosylated level, of either the core-fucosylated or outer arm fucosylated glycoforms can be a potential marker of HCC development.

In our present work, glycan and glycopeptides diagnostic ions produced by HCD and detected in DDA-MS/MS and PRM-MS/MS for glycopeptide identification and quantitative analysis, have been used to investigate the changes of glycosylation composition at individual glycosites in serum between patients with HCC and cirrhosis. This approach has served to provide an overview of N-glycopeptides as potential biomarker candidates for early-stage NASH HCC and subsequently allowed us to identify mid-abundance glycoproteins as potential markers directly from serum. Herein, we have studied 18 glycopeptide targets of CERU from 62 patient samples by LC-Stepped HCD-PRM-MS/MS and shown that the fucosylation ratio of a tri-antennary glycopeptide involving a glycosite at N762 is increased in patients with early HCC when compared to cirrhosis controls. This bi-fucosylated tri-antennary glycopeptide is core-fucosylated and contains an outer arm fucosylation site.

## Materials and Methods

### Materials

DTT (1,4-Dithiothreitol), IAA (Iodoacetamide) and 10 kDa MWCO Ultra-4 centrifugal filters were purchased from Sigma (St. Louis, MO). Human 14 Multiple Affinity Removal System Spin Cartridges (MARS) were from Agilent (Santa Clara, CA). The High pH Reversed-Phase Peptide Fractionation Kit (Thermo Scientific, Cat No.84868) and 7K MWCO Zeba Spin Desalting columns were from Thermo Scientific (Waltham, MA). The HILIC TopTips were purchased from Glygen (Columbia, MD). Sequencing-grade trypsin was purchased from Promega (Madison, WI).

### Serum Samples

All the serum samples from patients including 35 NASH cirrhosis 27 early-stage NASH HCC and 10 late-stage NASH HCC were from UT Southwestern Medical Center, Dallas, Texas, with institutional IRB approval. As described previously (23, 24), the clinical annotation of samples was obtained from a prospectively maintained database of treatment-naïve patients. NASH cirrhosis and NASH HCC were distinguished clinically by the presence of the metabolic syndrome, such as obesity, diabetes, and dyslipidemia, and absence of other causes of chronic liver disease, for example, viral hepatitis or alcohol abuse (25). HCC was defined per criteria from the American Association for the Study of Liver Disease, i.e. radiographically (arterial phase enhancement and delayed phase washout LIRADS 5 lesion), or histologically (26, 27). The clinical features of patients are listed in **Table 1**. All the serum samples were aliquoted and stored at −80°C. These 62 serum samples were classified as 2 groups: 35 NASH cirrhosis and 27 NASH early-stage HCC. Early-stage disease was defined by Milan Criteria, the most common criteria for liver transplantation in the United States. Power analysis was applied to analyze this classification by R studio (version 4.0.2) involving ‘pwr’ package.

**Table 1:** Clinical characteristics of individual patients with NASH for PRM analysis.

	Cirrhosis	Early HCC	Late HCC
No.	35	27	10
Gender (M/F)	11/24	15/12	4/6
Age (yrs)	58.6 (32-76)	68.0 (47-91.2)	67.8 (57-80.8)
Laboratory			
AFP (ng/mL)	3.7 (1.7-9.1)	32.4 (2-336)	10609.6 (12.9-37830)
TBili (mg/dL)	1.0 (0.2-3.3)	1.1 (0.2-3)	1.6 (0.3-3)
INR	1.1 (0.9-1.7)	1.2 (0.9-2.5)	1.1 (1.0-1.3)
Creatinine (mg/dL)	1.0 (0.5-7.4)	1.0 (0.5-2.5)	0.8 (0.4-1.3)
Score			
MELD	8.9 (6-23)	10 (6-21)	9 (7-12)
CTP	6 (5-8)	8 (5-10)	7 (5-10)
TNM stage % (I/II/III/IV)	NA	100/0/0/0	0/0/40/60
Ascites %	25.7%	40.7%	20%
Encephalopathy %	20%	22.2%	20%
AFP < 20 ng/mL	35 (100%)	23 (85.2%)	2 (20%)
Tumor size (cm)	NA	3.1 (1.3-6)	11.2 (4.6-18.5)

AFP, TBili, ALT, AST, INR, and creatinine values and MELD and CTP scores were provided by the UT Southwestern Medical Center. Values are presented as median with the interquartile range (IQR). AFP, alpha-fetoprotein; TBili, total bilirubin; ALT, alanine aminotransferase; AST, aspartate aminotransferase; INR, international normalized ratio; MELD, Model for end stage liver disease; CTP, Child-Turcotte-Pugh. N/A, Not Applicable.

### High Abundance Serum Proteins Depletion

**Figure 1:**
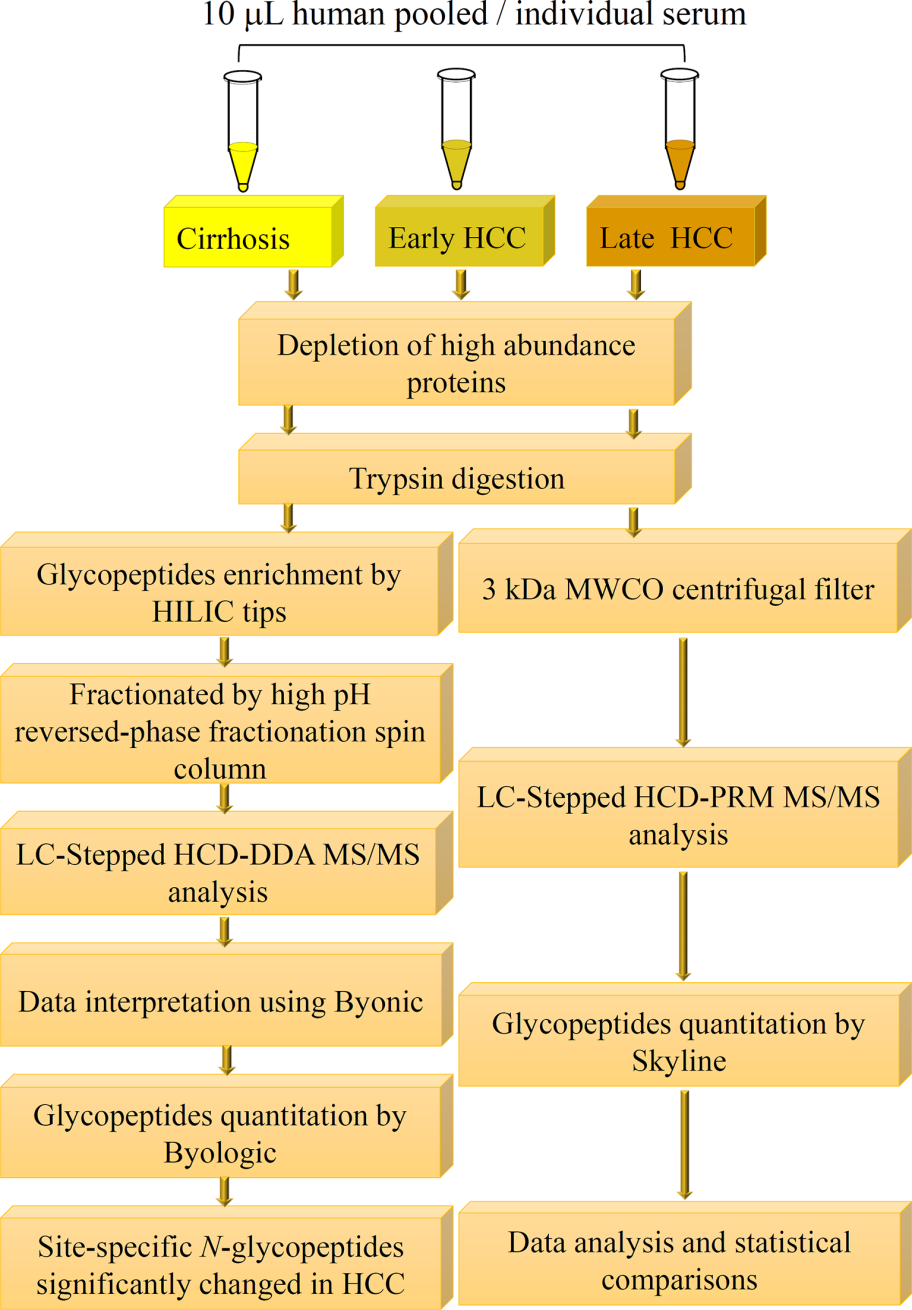
Workflow of quantitative LC-Stepped HCD analysis of intact N-glycopeptides derived from serum of patients between NASH-related liver cirrhosis, early-stage HCC, and late-stage HCC.

The workflow for initial screening for serum glycopeptides is outlined in **Figure 1**. 10 μL of pooled serum from 10 patients in each group, involving cirrhosis, early-stage NASH HCC, and late-stage NASH HCC, was diluted with 200 μL of PBS and filtered through a 0.22 μm filter according to the manufacturer’s instruction (6, 28). The filtered solution was loaded to the multiple affinity spin cartridge (Agilent, Santa Clara, CA) and centrifuged for 1.5 min at 100 × g at RT where the flow-through fraction was collected. Next, the cartridge was washed with 400 μL of washing buffer (buffer A, Agilent) twice by centrifugation for 2.5 min at 100 g at RT. These washed fractions were collected and combined with the flow-through fraction, which were then loaded to an Ultra-4 centrifugal filter for desalting and then dried down in a SpeedVac concentrator (Thermo).

### Protein Tryptic Digestion

The procedure of protein tryptic digestion was similar as our previous report (28). The depleted protein sample powder was dissolved by ABC buffer (ammonium bicarbonate, 50 mM, pH8.2). DTT (final concentration 20 mM) was added and incubated at 60°C for 30 min to reduce disulfide bonds, then, 50 mM IAA was added to alkylate the free thiol group for 30 min at RT. Then, 5 mM DTT was added to remove the extra IAA followed by a 7K MWCO Zeba™ Spin desalting column to remove the salts and dried down in a SpeedVac concentrator. The sample powder was re-dissolved in 50 mM ABC buffer where 3μL of trypsin (400 ng/μL) was added and incubated at 37°C for 14 hours for tryptic digestion. Finally, the digested products were dried down in a SpeedVac concentrator again for the following glycopeptide enrichment.

### Glycopeptide Enrichment

The tryptic digest was reconstituted in the binding buffer (15 mM ammonium acetate with 85% acetonitrile, pH3.5) and then loaded onto a pre-equilibrated HILIC tip where the glycopeptides were bound. After washing the HILIC tip 3 times with the binding buffer, the bound glycopeptides were released from the HILIC tip with water and then dried down in a SpeedVac concentrator (28).

### Off-Line Glycopeptide Fractionation for LC-Stepped HCD-DDA-MS/MS

The step glycopeptide fractionation method used was the same as in our previous report (28). The enriched glycopeptides were fractionated into eight fractions using a High pH Reversed-Phase Peptide Fractionation Kit. These dried glycopeptides were dissolved in 300 μL of 0.1% TFA solution, and then loaded onto the 0.1% TFA pre-conditioned column. After centrifugation at 3000 × g for 2 minutes, the eluate was kept as “flow-through” component. Then, 300 μL of water was loaded onto the column and centrifuged at 3000 × g for 2 minutes, the elution was kept as the “wash” component. These two components were left to confirm the binding efficiency of the column if needed. After these two steps, the targeted glycopeptides were eluted by an 8-fraction washing buffer of which the percentage of ACN was increased from 5% to 50% in 0.1% triethlyamine buffer. After the 8 fractionation steps, fractions 1 and 2 were combined and fractions 6, 7 and 8 were combined, considering there was limited material in these fractions, and all fractions were dried down in a SpeedVac for the following LC-MS/MS analysis.

### Glycopeptides Enrichment by Filter for LC-Stepped HCD-PRM-MS/MS

To achieve accurate quantitative analysis for the target glycopeptides from the serum samples, a simple approach of glycopeptides enrichment using a filter was performed for LC-Stepped HCD-PRM-MS/MS. This approach was adopted from the method for DDA-MS/MS. The difference was that the tryptic digested glycopeptides were enriched by a 3 kDa filter as well as by buffer exchange by distilled water five times using centrifugation (**Figure 1**). The glycopeptide solutions were then dried down in a SpeedVac concentrator.

### LC-Stepped HCD-DDA-MS/MS

The glycopeptides were dissolved in 0.1% formic acid (FA) and analyzed with duplicate injections on an Orbitrap Fusion™ Lumos™ Tribrid™ Mass Spectrometer (Thermo) coupled with a Dionex UPLC system. The binary solvent system included: phase A, 0.1% FA in H_2_O, and phase B, 80% CH_3_CN/0.1% FA. Glycopeptides were separated on a 75 μm × 50 cm column (C18, 2μm, 100 Å; Acclaim PepMap™ RSLC, Thermo) under a 90 min linear gradient from 2 to 40% phase B at a flow rate of 300 nL/min. The MS instrument was set in data dependent mode. The MS1 scans (*m/z* 400–1800) were acquired in the Orbitrap (120k resolution, 4e^5^ AGC, 100 ms injection time) followed by Stepped HCD MS/MS acquisition of the precursors with the highest charge states in order of intensity and detection in the Orbitrap (60k resolution, 2e^5^ AGC, 250 ms injection time). The Stepped HCD mode was operated with the stepped collision energies of 31.5%, 35%, and 38.5%, which was optimized for large-scale glycopeptide characterization based on our experimental process.

### LC-Stepped-HCD-PRM-MS/MS

The PRM-MS/MS method was adopted from a previous report (29). The LC-MS system was the same as that used in the DDA detection mode above. One difference is the gradient of the binary solvent where a 65 min linear gradient was used instead of the 90min gradient in the DDA mode. The gradient used was from 2 to 40% B at a flow rate of 300 nL/min. The stepped collision energies were set as 19%, 26% and 33% to fragment the glycopeptides. Another difference between the DDA and PRM analysis is that PRM required pre-defined precursor ion information, such as RT (retention time), MW (molecular weight), and charge states, whereas DDA-MS scans used all precursor ions. Under the PRM detection mode, the sensitivity of the detection is improved compared to the DDA detection mode. In our current experiment, to acquire the information of precursors, a survey scan was performed in DDA mode using a 65 min linear gradient, which was the same as in the PRM mode, before running the PRM analysis. Targeted glycopeptides were pre-defined as precursor ions, while the peptide backbone with one HexNAc moiety (pep+HexNAc) was defined as a Y1 ion for target quantitative analysis where Y1 has the strongest signal intensity among all Y ions in our present mass spectra (29). The targeted precursor ions were 18 glycopeptides from ceruloplasmin as listed in **Supplementary Table S1**.

### Data Interpretation and Relative Quantitation

For the DDA results, raw data were searched with Byonic (Protein Metrics), a software for peptide/glycopeptide and protein identification based on MS tandem spectra, and Proteome Discoverer 2.1 (Thermo Fisher Scientific, San Jose, CA) software using the Byonic (Protein Metrics) search engine (30). A UniProt human protein database which includes 20359 proteins was used for data searching. The searching parameters were set as (1): fixed modification, carbamidomethyl (C); (2) variable modifications, oxidation (M); deamidation (N, Q) and N-glycan modifications (N); (3) 1 missed cleavage; (4) mass tolerance, 10 ppm for MS1 and 20 ppm for MS2. The searching results were filtered at a confidence threshold of Byonic score > 150, Delta modification score > 10, PEP2D < 0.05, and FDR2D < 0.01. The theoretical m/z of the oxonium ions from HexNAc (m/z 138.05, m/z 168.05 and m/z 204.09), NeuAc (m/z 274.09 and 292.10), HexNAcHex (m/z 366.14), HexHexNAcFuc (m/z 512.20) and HexNAcHexNeuAc (m/z 657.23) in glycopeptides from HCD-MS are known for glycan identification (20, 30). These oxonium ions were also checked manually.

For the DDA results, quantitative analysis was also performed using Byologic as described previously (20, 30). With Byologic, the peak area of the XIC of a given glycopeptide was automatically integrated and normalized against the sum of peak areas of all glycopeptides identified in each MS run, providing a relative quantitation of each N-glycopeptide in the sample. The relative abundance of a glycopeptide was calculated by the sum of the glycopeptide bearing the same glycan at the glycosite with different charges, and the results were exported to an EXCEL workbook.

For the replication experiment analysis, the normalized and integrated data of triplicate experiments were converted to csv format file, and the Pearson correlation coefficient was presented as a heatmap by R studio (version 4.0.2).

For the PRM results, we used the Skyline software for quantification of the selected glycopeptides. Similar to DDA analysis, oxonium ions HexNAc, NeuAc, HexNAcHex, HexHexNAcFuc and HexNAcHexNeuAc and other possible b/y ions were used for glycopeptide identification, while the Y1 ion (peptide+HexNAc) was used for quantification. Peptide settings and transition settings were required when performing the Skyline analysis. Of the peptide settings, a library (.ssl file) involving the information of the precursor ions was created with the parameters retrieved from a survey scan before PRM detection, including glycopeptide sequence, scan number, retention time, charges. A.ms2 file converted from the survey scan raw data was also required. The sequence of the targeted proteins was uploaded as the background protein database. Of the transition settings, the precursor charges were set as +1 to +6, the ion charges were set as +1 and +2, and the ion types were set as y and b ions. The ion match tolerance was set as 0.05 m/z. The Y1 ions from the targeted glycopeptides were referred as (peptide +HexNAc). The integral peak areas of the Y1 ions were exported from Skyline in.csv format, which were used for glycopeptide quantification and data normalization. The relative abundance of each glycopeptide was calculated by normalizing its peak area to the sum of peak areas of all targeted glycopeptides in each sample (28, 29). Next, the relative abundances of the target glycopeptides were compared between different disease state groups and the scatter plots were created by GraphPad PRISM software (version 8.0). The fucosylation ratio was calculated as follows:


$$\begin{array}{c} \text{fucosylation ratio}=\text{area (XIC of fucosylated peptide)/}\\ \text{area}(\text{XIC of corresponding non}-\text{fucosylated peptide}) \end{array}$$

## Results

### Serum Samples Classification and Power Analysis

A power analysis was applied to the sample groups before our experiment. The power value in this analysis is 0.87 when the parameters were set as follows: sample sizes were 35 for NASH cirrhosis versus 27 early-stage NASH HCC, the effect size was d = 0.8, and significance level was set at 0.05. The effect size set as d = 0.8 was considered reasonable based on prior studies (31). and power of 0.87 indicated that the sample size in this experiment was acceptable.

### Workflow for LC-Stepped-HCD -DDA -MS/MS and LC-Stepped-HCD-PRM-MS/MS Analyses

**Figure 2:**
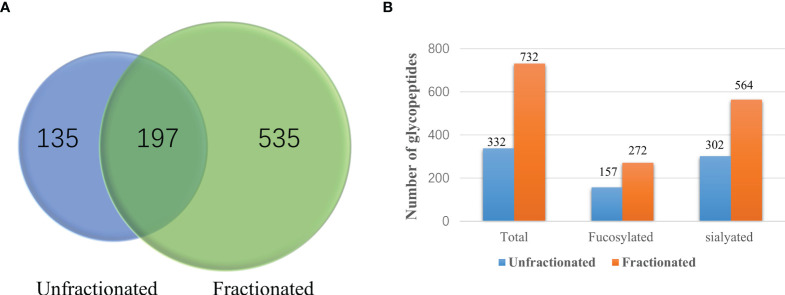
Comparisons of the number of unfractionated and fractionated glycopeptides from pooled late-stage HCC samples. **(A)** Venn diagram showing the number of glycopeptides from unfractionated and fractionated glycopeptides. **(B)** Comparison of the number of total glycopeptides, fucosylated glycopeptides and sialylated glycopeptides between the unfractionated and fractionated groups.

As shown in **Figure 1**, we have established a workflow for large-scale intact site-specific glycopeptide identification from serum samples, which included depletion of high-abundance proteins, enrichment of glycopeptides, offline peptide fractionation, and LC-stepped HCD-DDA-MS/MS for differential determination of glycosylation composition changes in patient sera between different disease states. The detection of low abundance glycopeptides in complex mixtures in DDA-MS is quite difficult due to the mass spectral signals where the glycopeptides can be suppressed by non-glycopeptides. To solve this problem, highly efficient removal of the high abundance proteins from serum samples and enrichment of the tryptic digested glycopeptides is required. The HILIC-based glycopeptide enrichment approach has been widely used in glycan/glycopeptide research (13). Also, it was found that fractionation of the glycopeptides by pH and hydrophobicity was helpful for glycopeptide biomarker discovery, where the number of glycopeptides increased after fractionation (**Figure 2A**) (28, 32).

MS data acquired in DDA mode is an appropriate means for broadscale screening of glycopeptides. However, the accuracy is not sufficient for quantitative analysis of the target glycopeptides in DDA-MS/MS. To further quantitatively analyze these glycopeptides, we established a workflow for quantitative analysis of glycopeptides by LC-stepped-HCD-PRM-MS/MS as shown in **Figure 1**. To minimize the number of sample operation steps and improve the accuracy of quantification, tryptic digestion of samples was followed by only one single step of a 3 kDa MWCO filter for glycopeptide enrichment and desalting before PRM-MS/MS analysis.

### HILIC Enrichment and Fractionation Are Essential for Glycopeptide Biomarker Candidate Discovery From Pooled Serum Samples for DDA-MS/MS Detection

In our present work, we first compared the results from unfractionated and fractionated glycopeptides after HILIC enrichment. A Venn diagram of the unfractionated and fractionated samples are shown in **Figure 2A**. It was clear that more glycopeptides were observed from the fractionated samples compared to the unfractionated group. We also compared the number of fucosylated and sialylated glycopeptides from these two methods. Using the fractionation method, we observed 272 fucosylated and 564 sialylated glycopeptides from a total of 732 glycopeptides as compared to 157 fucosylated and 302 sialylated glycopeptides from a total of 332 glycopeptides without using fractionation (**Figure 2B** and **Supplementary Table S2**).

### Reproducibility of the Workflow

**Figure 3:**
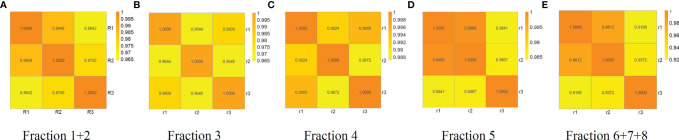
Reproducibility analysis of three independent replicates of fraction 1 + 2, 3, 4, 5 and 6 + 7+8 from a random serum sample. Pearson correlation coefficient R^2^ values for the binary comparison of the three replicates range from 0.9195 to 0.9989, demonstrating good reproducibility of the method. **(A)** fraction1+2, **(B)** fraction 3, **(C)** fraction 4, **(D)** fraction 5, and **(E)** fraction 6 + 7+8.

The reproducibility of the workflow was evaluated using the percentage of each glycopeptide from each run by three independent experiments. The Pearson correlation coefficient R^2^ values for the binary comparison of the three replicates from 0.9195 to 0.9989 is shown in **Figure 3**.

### Comparisons of Glyco-Distributions in Different Stages of HCC From Pooled Samples

**Figure 4:**
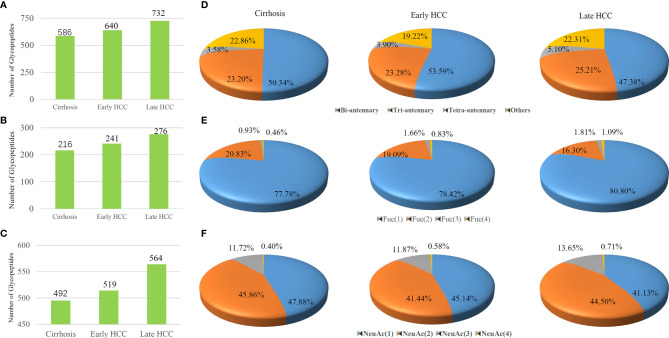
Comparisons of the **(A)** total glycopeptides, **(B)** fucosylated glycopeptides and **(C)** sialylated glycopeptides among cirrhosis, early-stage HCC and late-stage HCC samples are represented by columns. Distribution of glycopeptides in different **(D)** antennas, **(E)** fucosylated glycan numbers and **(F)** sialylated glycan numbers are represented by pie charts..

We studied the various site-specific glyco-distribution of serum glycoproteins from different stages of HCC and were able to compare them based on the percentage of different fucosylated and sialylated glycopeptides (**Figure 4**). In our present work, the LC-stepped HCD-DDA-MS/MS analysis of the pooled samples resulted in the identification of 586 glycopeptides in cirrhosis, 640 glycopeptides in early-stage NASH HCC, and 732 glycopeptides in late-stage NASH HCC (**Figure 4A**). Of these glycopeptides, nearly half are of bi-antennary glycoform in each stage of HCC (50.34% in cirrhosis, 53.59% in early-stage HCC and 47.38% in late-stage HCC) (**Figure 4D**). As shown in **Figure 4B**, there were 216, 241 and 276 corresponding to cirrhosis, early-stage HCC and late-stage HCC, respectively. Of these fucosylated glycopeptides, mono- fucosylated glycopeptides changed from 77.78% in cirrhosis to 80.80% in late-stage HCC. The tri-fucosylated glycopeptides varied from 0.93% to 1.81% during the progression from cirrhosis to late-stage HCC. Similarly, the percentage of tetra-fucosylation was also increased during disease progression, although only detected at 0.46% to 1.09%.

We also investigated the changes of sialylated glycopeptide levels during HCC progression. As shown in **Figure 4C**, the total number of sialylated glycopeptides were 492, 519 and 564 corresponding to cirrhosis, early-stage HCC and late-stage HCC. **Figure 4F** shows the percentages of sialylated glycopeptides among the HCC stages. Both the mono- and di-sialylated were about 41~ 48%, occupying the majority of sialylated glycoforms. Although the trend of percentages of mono- and di-sialylated glycopeptides decreased during the progression of HCC, the tri-and tetra-sialylated glycopeptides increased from cirrhosis to HCC where an increase of 11.72% to 13.65% and 0.40% to 0.71% for tri-and tetra-sialylated glycopeptides, were observed respectively. In general, the proportion of fucosylated and sialylated glycopeptides increases during the progression of disease (33).

### The Ratio of Fucosylated Glycopeptides From Ceruloplasmin Identified as Potential Biomarker Candidates by LC-Stepped-HCD-PRM-MS/MS

Previous reports demonstrated that high levels of fucosylated and sialylated glycopeptides were related to the progression of HCC (16, 17, 22, 33–37). It has been shown that core-fucosylation ratios of ceruloplasmin were increased during the progression of alcohol related HCC as well as the HBV related HCC after Endo F3 partially truncated the glycan structure of glycopeptides. Our present results from pooled samples showed that fucosylated tri-antennary glycopeptides from ceruloplasmin (CERU) can be detected in cirrhosis and late-stage HCC in DDA-MS/MS. Combining all results above, LC-Stepped HCD-PRM MS/MS was further performed for targeted quantitative analysis of the selected glycopeptide candidates from CERU among individual patients to validate the differential expression of fucosylated glycopeptides. 18 site-specific glycopeptides were selected for PRM analysis (**Supplementary Table S1**). Of the total 62 patient samples including 35 cirrhosis, 27 early-stage HCC, we quantitatively analyzed the PRM results of the 18 targeted glycopeptides by Skyline software. These 18 glycopeptides were from 4 CERU glycosites (site138, 358, 397 and 762), and based on the glycopeptides can be detected by DDA MS/MS from pooled samples. The XIC values were exported from the Skyline platform as a.csv workbook and were normalized. The relative abundance of the glycopeptides was compared by *t*-test.

**Figure 5:**
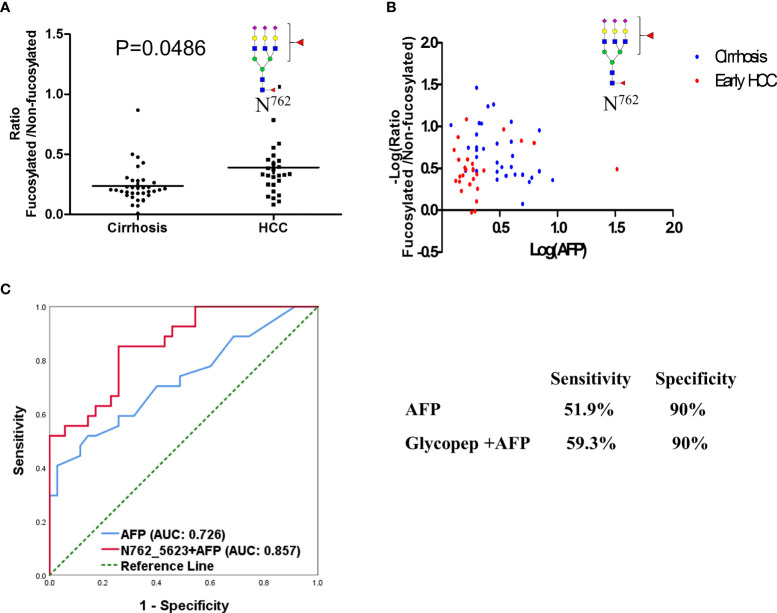
Fucosylation ratio of glycopeptide **(A)** ELHHLQEQN^762^VSNAFLDK_HexNAc(5)Hex(6)Fuc(2)NeuAc(3)of ceruloplasmin in cirrhosis and early stage HCC serum samples. **(B)** Two-dimensional plot of AFP and fucosylation ratio of this glycopeptide between cirrhosis and HCC samples. **(C)** ROC analysis of glycopeptide in combination with AFP in distinguishing HCC from cirrhosis.

In our present study, the ratio of fucosylated tri-antennary glycopeptides from CERU site N762 was increased significantly from cirrhosis to early HCC (P<0.05) (**Figure 5A**). Two-dimensional plots of AFP and the fucosylation ratio of this glycopeptide is shown in **Figures 5B**, which indicates the relationship of the fucosylation ratio of this glycopeptide and the concentration of AFP in cirrhosis and early-stage HCC. The relative abundance of the glycopeptide involving site N762 had no significant changes from cirrhosis to early HCC (**Supplementary Figures S1A–E**). and the ratio of these glycopeptides are shown in **Supplementary Figures S1F, G**. We believe that the ratio of fucosylated glycopeptides can reflect the fucosylation changes better than the abundance changes in individual samples as the ratio considers the non-fucosylated glycopeptides as a background in individual samples. The receiver operating characteristic curve (ROC) analysis of glycopeptide ELHHLQEQN^762^VSNAFLDK_HexNAc(5)Hex(6)Fuc(2)NeuAc(3) is shown in **Figure 5C**, where the area under the receiver operating characteristic curve (AUC) of AFP alone is 0.726, whereas a combined analysis with AFP results in an AUC of 0.857.

**Figure 6:**
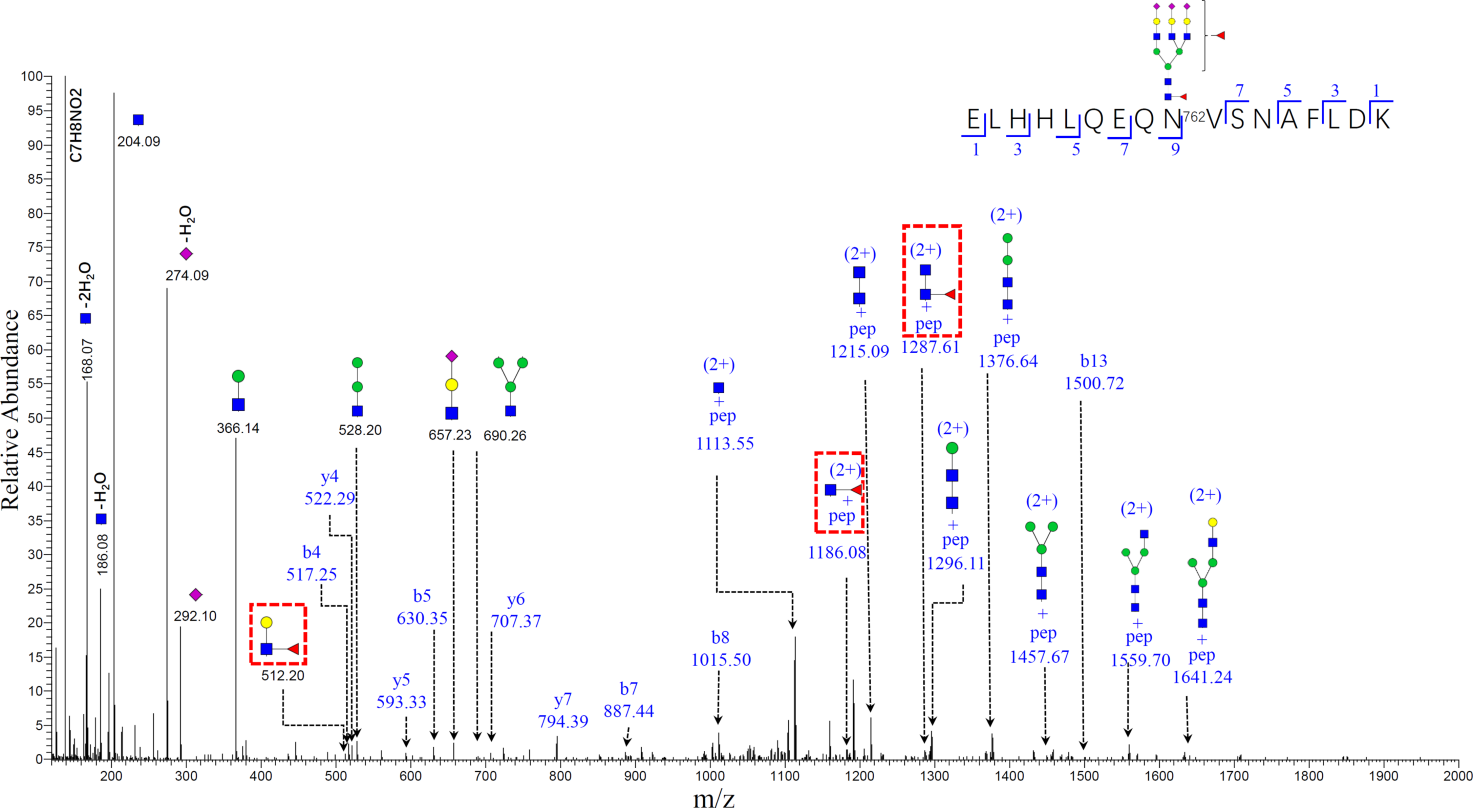
Representative MS/MS spectrum of N-glycopeptide of ELHHLQEQN^756^VSNAFLDK with the glycan HexNAc(5)Hex(6)Fuc(2)NeuAc(3). The oxonium ions, glycosidic fragments and b/y fragments from the peptide backbone were well characterized. Specific diagnostic fragment ion (HexNac+Gal+Fuc) at m/z 512.20 confirmed the outer-arm fucosylation, and ions (pep+HexNac+Fuc) at m/z 1186.08 and (pep+2HexNac+Fuc) at m/z 1287.61 (marked by red dashed rectangles) confirmed the core-fucosylation. (The symbols used in the structural formulas: blue square = HexNAc; green circle = Man; yellow circle = Gal; purple diamond = NeuAc).

The mass spectrum of the bi-fucosylated glycopeptide involving site 762 ELHHLQEQN^762^VSNAFLDK_HexNAc(5)Hex(6)Fuc(2)NeuAc(3) is shown in **Figure 6**. The doubly-charged diagnostic ions at m/z 1186.08 (peptide + HexNac + Fuc) and m/z 1287.61 (peptide+2HexNac+Fuc) confirmed it was a core-fucosylated tri-antennary glycopeptide. In addition to these two diagnostic ions, another diagnostic ion at m/z 512.20 (HexNac + Glc + Fuc) can be detected, which suggests the second fucosylated site was outer-arm in this bi-fucosylated tri-antennary glycopeptide. However, there were no fucose containing diagnostic ions in the mass spectrum of ELHHLQEQN^762^VSNAFLDK_HexNAc(5)Hex(6)NeuAc(3) (**Supplementary Figure S2**). The relative abundance of the glycopeptide involving site N762 is shown in (**Supplementary Figure S1**). The relative abundance of bi-antennary glycopeptides and the non-fucosylated tri-antennary glycopeptides had no significant changes from cirrhosis to early HCC. The ratio of the fucosylated bi-antennary glycopeptide involving this site had no significant increase either. The other relative abundance and ratio of fucosylated to non-fucosylated glycopeptides involving sites N138, N358, and N397 are shown in **Supplementary Figures S3**–**S5**. As shown in **Supplementary Figure S3**, the relative abundance of these nonfucosylated or fucosylated bi-and tri- antennary glycopeptides in site N138 had no significant changes in the progression from cirrhosis to early HCC (P>0.05) (**Supplementary Figures S3A–G**). The ratio of fucosylated to non-fucosylated forms of these mono-fucosylated bi-antennary and bi-fucosylated tri-antennary glycopeptides is shown in **Supplementary Figures S3H, I**. Their ratio had no significant changes when the P value is larger than 0.05.

Similarly, the relative abundance and the ratio of fucosylated to non-fucosylated glycopeptides involving site N397 of cirrhosis and early-stage HCC is shown in **Supplementary Figure S4**. It was clear that there were no distinguishing differences between the cirrhosis and early-stage, considering the relative abundance or ratio of fucosylation of these glycopeptides.

Finally, we also detected two non-fucosylated glycopeptides involving site N358 **Supplementary Figure S5**. Of these two non-fucosylated glycopeptides, the bi-antennary glycopeptide had no significant increase from cirrhosis to early HCC. We have no information on the fucosylation ratio since the corresponding fucosylated glycopeptide was not acquired in DDA-MS/MS.

## Discussion

We have established a workflow for large-scale scanning of glycopeptides from serum pooled samples using DDA-MS/MS, which included depletion of high abundance serum proteins, enrichment of glycopeptides by HILIC-based cartridges and pH and hydrophobicity fractionation. These steps improved the mass spectrometry spectra signal intensity of glycopeptides based on: (1) the signal of glycopeptides, especially the glycopeptides from middle and low abundance glycoproteins, which would otherwise be suppressed when the non-glycopeptides co-elute with glycopeptides. The step of depletion of high abundance serum overcomes this issue; (2), the widely used HILIC-based glycopeptide enrichment approach which has been involved in the present workflow.

Recently, our group reported the characterization of site-specific glycopeptides enriched from human serum haptoglobin digests using HILIC TopTips as analyzed by LC-EThcD-MS/MS (30) which has been widely used in intact sialylated glycopeptides and phosphorylated glycopeptides fragmentation (38, 39), as well as vitronectin by LC-HCD-MS/MS on an Orbitrap Fusion Lumos Tribrid mass spectrometer (28). Herein, we combined the depletion of the high abundance proteins, HILIC based glycopeptide extraction and fractionation methods to improve the MS signal of glycopeptides, particularly low abundance proteins in serum samples, prior to MS (40). Thus, we used this enrichment method to enhance the MS signals of glycopeptides, particularly the low abundance glycopeptides. Also, the total number of glycopeptides were increased after the samples were fractionated as compared to the unfractionated group (**Figure 2A**). Using the fractionation method, 272 fucosylated glycopeptides can be detected compared to 157 fucosylated glycopeptides without fractionation. Furthermore, the sialylated glycopeptides also increased from 302 to 564 after fractionation. These results demonstrated that the fractionation step was critical to improve the detection of glycopeptides, especially the low abundance glycopeptides in complex mixtures. (**Figure 2B** and **Table S2**). The reproducibility of the workflow was evaluated by three independent experiments. The Pearson correlation coefficient R^2^ values for the binary comparison of the three replicates were more than 0.91, which demonstrate a good reproducibility of the workflow.

In order to investigate the glycosylation changes during the progression of HCC, we made comparisons of antenna number, fucosylation and sialylation levels among the different disease stages. Herein, pooled samples were used to understand the overall distribution of glycopeptides in the different disease groups and was a time- and cost-effective method as compared to analyzing individual samples. The results demonstrated that the number of the total glycopeptides increased from 586 to 732 during HCC progression as shown in **Figure 4A**, which was consistent with previous reports that glycopeptides increased in advanced HCC stage (33). We observed that the percentage of bi-antennary glycopeptides was in the range of 47.38% to 53.59% in the disease stages.

We also observed that the percentage of tri-antennary and tetra-antennary glycopeptides increased during the progression of cirrhosis to late-stage HCC. Recent work has reported that highly fucosylated or/and sialylated glycopeptides were related to the progression of HCC (16, 22, 28, 29, 34). The fucosylated glycopeptides are shown in **Figure 4B**. The total number of fucosylated glycopeptides increased from 216 to 276 during the progression from cirrhosis to late-stage HCC. Of the fucosylated glycopeptides, the mono-fucosylated glycopeptides had the highest proportion of 77.78% to 80.80% in different disease stages. It is noteworthy that the percentage of multi-fucosylated glycopeptides bearing three or four fucoses increased from cirrhosis to late-stage HCC. Core-fucosylated glycopeptides from several proteins such as CERU have been reported as biomarker candidates in HCC after the glycan structure was partially removed by Endo F3 (16). Also, haptoglobin has been noted by its multi-fucosylated glycoforms (20, 21, 30, 41). The presence of sialylated glycopeptides are also notable. The total number of sialylated glycopeptides increased from 492 to 564 during the progression of cirrhosis to late-stage HCC. This was consistent with previous reports on sialylated glycopeptides increasing in advanced HCC stage. We also observed that the tri-and tetra-sialylated glycopeptides increased from cirrhosis to HCC, which further confirmed that the tri- and tetra-antennary glycopeptides increased in late-stage HCC as the sialic acids are usually located at the end of glycan antennas. As discussed above, these results suggested that the total glycopeptides, the fucosylated glycopeptides, and sialylated glycopeptides increased during HCC progression. The percentage of tri-and tetra-antennary glycan forms and multi-fucosylated glycopeptides also increased in this progression.

Our previous reports demonstrated that the core-fucosylated glycopeptides from CERU increased during the progression of alcohol related HCC and HBV related HCC after Endo F3 partial removal of the glycan structure of glycopeptides. In the current work, PRM-MS/MS was performed for targeted quantitative analysis of the selected glycopeptide candidates from CERU among individual patients, to validate the differential expression of fucosylated glycopeptides. Using PRM-MS/MS, we can obtain the glycan form information from glycopeptides instead of only the core-fucosylated structure which remains after glycan partial removal by Endo F3.

In our current experiment, 18 site-specific glycopeptides were selected for PRM-MS/MS analysis based on our DDA-MS/MS results from pooled samples and our previous reports (16). There were a total 62 patient samples, including 35 cirrhosis and 27 early-stage HCC analyzed. The sample size is sufficient for this biomarker study as confirmed by power analysis (power = 0.87 when the significant level was set as 0.05). Considering individual differences, we used the ratio of fucosylated glycopeptides to show the fucosylation changes instead of relative abundance. At the site N762 as shown in **Figures 5A**, the fucosylation ratio of the bi-fucosylated tri-antennary glycopeptide involving this site increased significantly from cirrhosis to early-stage HCC. The P values of the fucosylation ratio of ELHHLQEQN^762^VSNAFLDK_HexNAc(5)Hex(6)Fuc(2)NeuAc(3) was 0.0486. This site had been reported by our group as a potential marker in alcohol-related HCC (16), but the use of EndoF3 eliminates the outer-arm fucosylation glycoform information.

An advantage for mass spectrometry is that it can reveal the subtle changes of glycoforms even though they are attached to the same peptide backbone. The doubly charged diagnostic ions at m/z 1287.61 and m/z 1186.08 in **Figure 6** confirmed that it is a core-fucosylated glycopeptide. Further, the bi-fucosylated tri-antennary glycopeptide involving site 762 contained an outer arm fucose as the diagnostic ion at m/z 512.20 (HexNAc+ Hex+Fuc). The core-fucosylated glycopeptide involving this site also has been reported by our previous work as a potential marker in alcohol-related HCC, hepatitis B virus (HBV), and hepatitis C virus (HCV) (16).

In previous work, Tan et al. (6) used Lens culinaris Agglutinin (LCA) to enrich the fucosylated glycopeptides digested from a 13 pancreatic cancer serum sample set and found that the core-fucosylated glycopeptide involving this site increased from the control group to chronic pancreatitis. Thus, we speculate that core-fucosylation of this site may be related to the development of a variety of tumors, not only HCC. We also examined the relationships between the fucosylation ratio of this glycopeptide and the AFP values by 2D-plots as shown in **Figures 5B**, in which the X axis is the value of Log (AFP) calculated from clinical results and Y axis is –Log (fucosylation ratio). Here we use “minus log” due to fucosylation ratios being less than 1 in most cases. The receiver operating characteristic curve (ROC) analysis of this glycopeptide combined with AFP compared to AFP alone demonstrated that the AUC of the glycopeptide combined with AFP outperformed AFP only as shown in **Figure 5C** The AUC of glycopeptide ELHHLQEQN^762^VSNAFLDK_HexNAc(5)Hex(6)Fuc(2)NeuAc(3) resulted in a significant improvement achieving an AUC of 0.857 when combined with AFP, whereas a AUC of 0.726 was obtained for AFP only. When the specificity was set at 90%, the sensitivity of this glycopeptide combined with AFP is 59.3% for ELHHLQEQN^762^VSNAFLDK_HexNAc(5)Hex(6)Fuc(2)NeuAc(3), whereas the sensitivity is 51.9% for AFP only. These results indicate that even on the same glycoprotein, there are different fucosylated levels in different glycosylation sites In addition, this glycopeptide has three sialic acids where this result is consistent with previous reports that the fucosylated or/and sialylated glycopeptides from CERU were related to the progression of HCC (16, 17, 22, 33–35).

## Conclusions

We have established a workflow based on LC-Stepped HCD-DDA-MS/MS for screening site-specific N-glycopeptide biomarkers from human sera for NASH HCC. In this workflow, a procedure was optimized including removal of the top 14 high abundance serum proteins, HILIC enrichment of glycopeptides, and fractionation of glycopeptides, prior to mass spectrometry analysis. This approach was evaluated by three independent experiments from serum samples and the Pearson correlation coefficient R^2^ values for the binary comparison of the three replicates which showed an excellent reproducibility of the workflow. From the DDA-MS/MS results and in combination with previous reports, 18 glycopeptides from CERU were selected and analyzed in 35 cirrhosis and 27 early-stage HCC samples by LC-Stepped HCD-PRM-MS/MS to further investigate the fucosylation ratio of these glycopeptides. Of these glycopeptides, the ratio of a bi-fucosylated tri-antennary glycopeptide involving site N762, ELHHLQEQN^762^VSNAFLDK_HexNAc(5)Hex(6)Fuc(2)NeuAc(3) increased from cirrhosis to early-stage HCC. This fucosylated glycopeptide is core-fucosylated, and contained an outer-arm fucose. These results indicate that this bi-fucosylated tri-antennary glycopeptide in CERU could be a potential biomarker for further study and validated in a larger sample set and should be a promising candidate for early detection of NASH HCC.

## Data Availability Statement

The datasets presented in this study can be found in online repositories. The names of the repository/repositories and accession number(s) can be found in the article/**Supplementary Material**.

## Ethics Statement

The studies involving human participants were reviewed and approved by the University of Texas Southwestern IRB and the University of Michigan IRB. The patients/participants provided written informed consent to participate in this study. Samples and data were shared using a material transfer and data use agreements between the Universities.

## Author Contributions

YL and DL designed the experiment. YL performed the experiments, MS analysis and statistical analysis with the help of JZ. YL wrote the manuscript. AA, AS, and NP collected the samples and clinical information. NP and DL edited the manuscript. All the authors contributed to manuscript review. All authors contributed to the article and approved the submitted version.

## Conflict of Interest

The authors declare the following competing financial interest(s): AS serves as a consultant to Glycotest, Exact Sciences, Roche, Bayer, and GRAIL and has served on advisory boards for Fujifilm Medical Sciences and Exact Sciences. NP serves as a consultant for Exact Sciences, Bayer, Eli Lilly, and Bristol Myers-Squibb, has served on advisory boards for Wako/Fujifilm and Genentech and received institutional research funding from Glycotest, Exact Sciences, TARGET RWE, and Bayer.

The remaining authors declare that the research was conducted in the absence of any commercial or financial relationships that could be construed as a potential conflict of interest.

## Publisher’s Note

All claims expressed in this article are solely those of the authors and do not necessarily represent those of their affiliated organizations, or those of the publisher, the editors and the reviewers. Any product that may be evaluated in this article, or claim that may be made by its manufacturer, is not guaranteed or endorsed by the publisher.

## Supplementary Material

Supplementary Figure S1 Relative abundance of bi- and tri-antennary glycopeptides ELHHLQEQN^762^VSNAFLDK of ceruloplasmin in cirrhosis and early-stage HCC serum samples **(A–E)**. Ratio of fucosylated to non-fucosylated forms of these glycopeptides **(F)** and **(G)**.

Supplementary Figure S2 Representative MS/MS spectrum of N-glycopeptides of ELHHLQEQN^756^VSNAFLDK with the glycan HexNAc(5)Hex(6)NeuAc(3). The oxonium ions, glycosidic fragments and b/y fragments from the peptide backbone were well characterized. (The symbols used in the structural formulas: blue square = HexNAc; green circle = Man; yellow circle = Gal; purple diamond = NeuAc).

Supplementary Figure S3 Relative abundance of bi-, tri- and tetra-antennary glycopeptides EHEGAIYPDN^138^TTDFQR of ceruloplasmin in cirrhosis and early-stage HCC serum samples **(A–G)**. Ratio of fucosylated to non-fucosylated form of these glycopeptides **(H, I)**.

Supplementary Figure S4 Relative abundance of bi- and tri-antennary glycopeptides EN^397^LTAPGSDSAVFFEQGTTR of ceruloplasmin in cirrhosis and early-stage HCC serum samples **(A–E)**. Ratio of fucosylated to non-fucosylated forms of these glycopeptides **(F–H)**.

Supplementary Figure S5 Relative abundance of bi- and tri-antennary glycopeptides AGLQAFFQVQECN^358^K of ceruloplasmin in cirrhosis and early-stage HCC serum samples **(A, B).**

Supplementary Table S1 PRM precursor ions list of ceruloplasmin.

Supplementary Table S2 Comparisons of the number of unfractionated and fractionated glycopeptides from pooled late-stage HCC samples.

Supplementary Table S3 Distribution of glycopeptides in different antennas, fucosylated and sialylated glycan numbers in cirrhosis.

Supplementary Table S4 Distribution of glycopeptides in different antennas, fucosylated and sialylated glycan numbers in early-stage HCC.

Supplementary Table S5 Distribution of glycopeptides in different antennas, fucosylated and sialylated glycan numbers in late-stage HCC.
