# Supplementary figures and images for: A Fucosylated Glycopeptide as a Candidate Biomarker for Early Diagnosis of NASH Hepatocellular Carcinoma Using a Stepped HCD Method and PRM Evaluation
Source: Front Oncol. 2022 Mar 17;12:818001. doi: 10.3389/fonc.2022.818001 (PMC8970044; doi:10.3389/fonc.2022.818001)

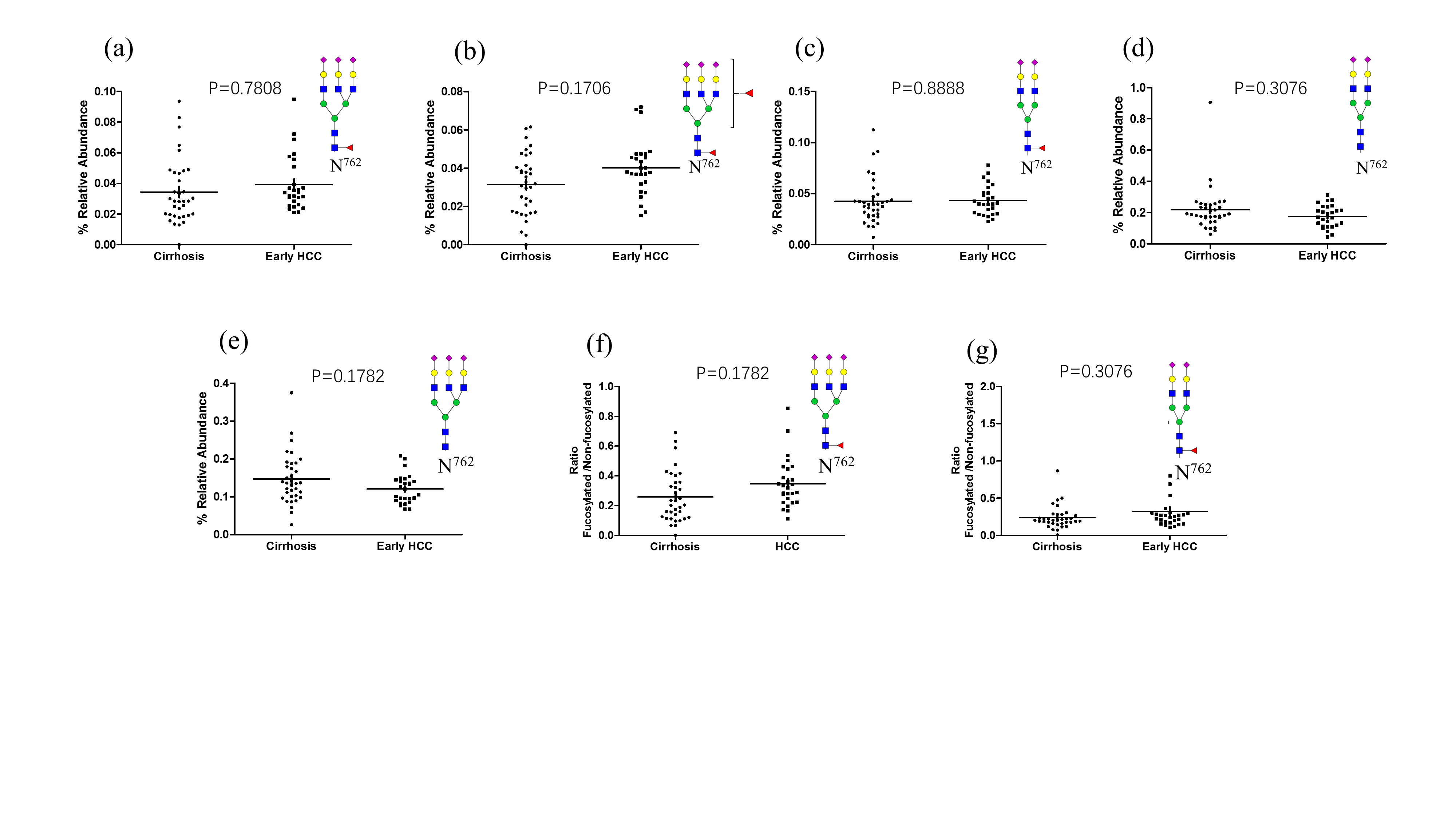

Supplement: Supplementary Figure S1 — Relative abundance of bi- and tri-antennary glycopeptides ELHHLQEQN762VSNAFLDK of ceruloplasmin in cirrhosis and early-stage HCC serum samples (A–E). Ratio of fucosylated to non-fucosylated forms of these glycopeptides (F) and (G). [file Image_1.tif]

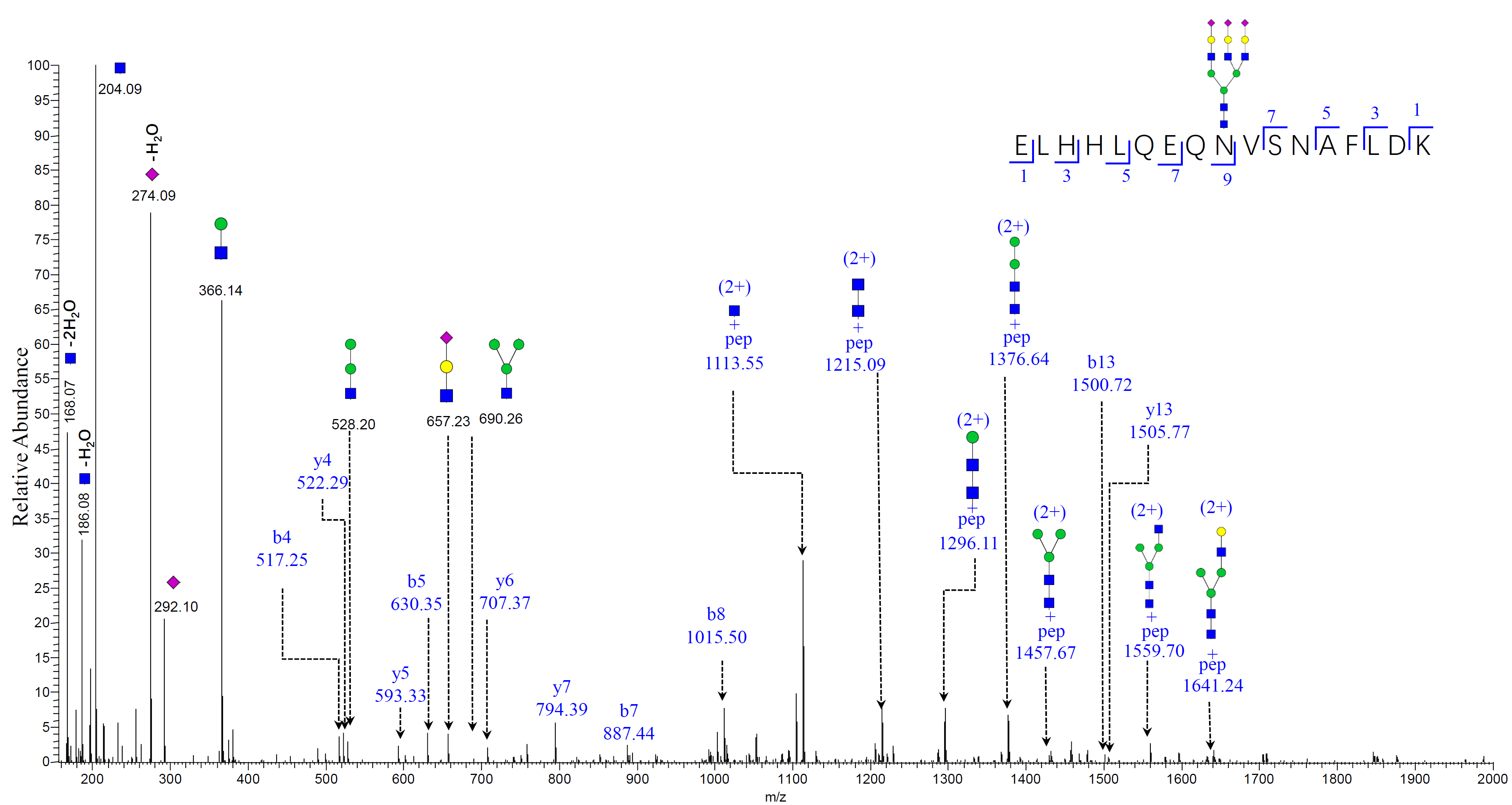

Supplement: Supplementary Figure S2 — Representative MS/MS spectrum of N-glycopeptides of ELHHLQEQN756VSNAFLDK with the glycan HexNAc(5)Hex(6)NeuAc(3). The oxonium ions, glycosidic fragments and b/y fragments from the peptide backbone were well characterized. (The symbols used in the structural formulas: blue square = HexNAc; green circle = Man; yellow circle = Gal; purple diamond = NeuAc). [file Image_2.tif]

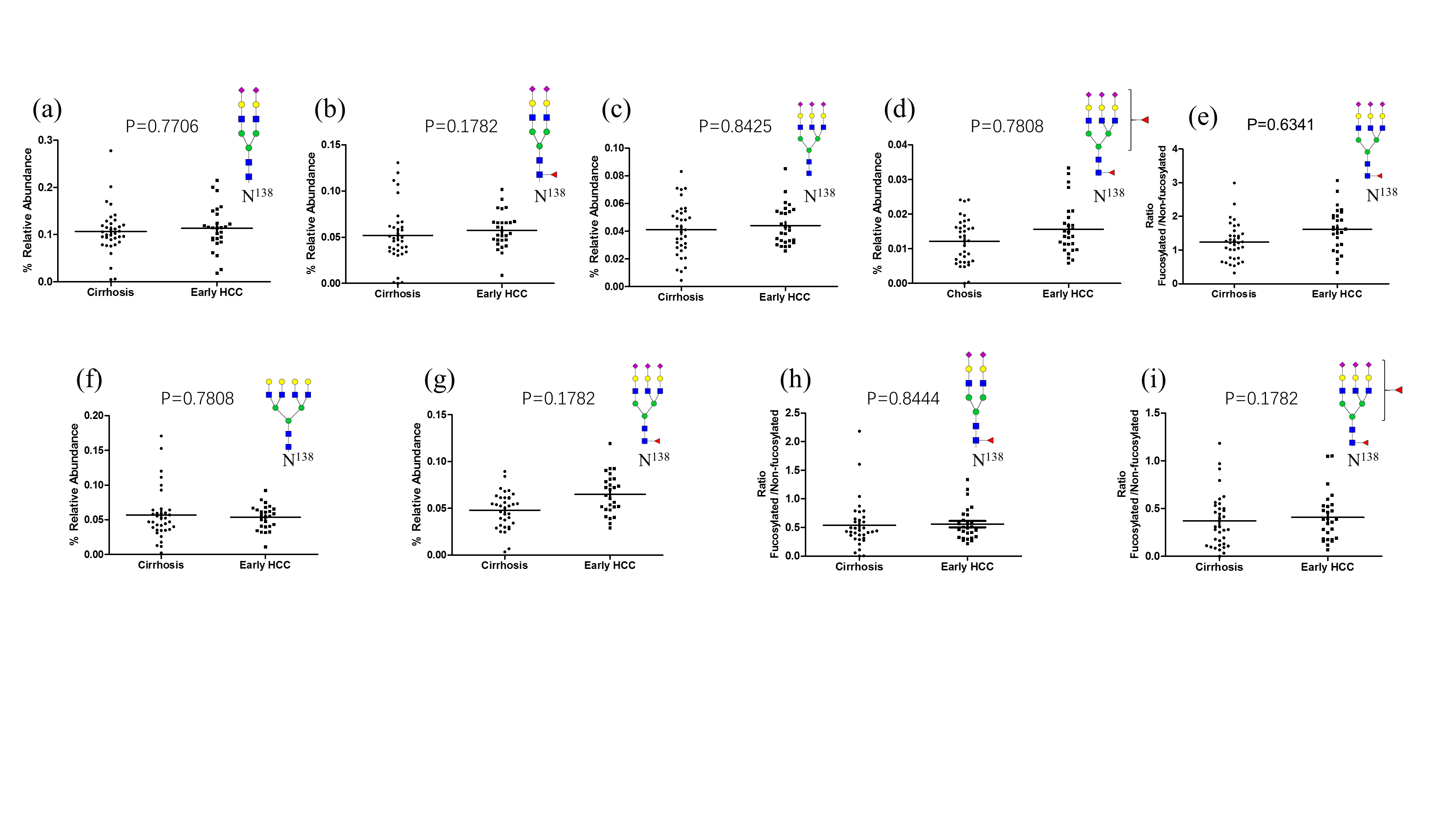

Supplement: Supplementary Figure S3 — Relative abundance of bi-, tri- and tetra-antennary glycopeptides EHEGAIYPDN138TTDFQR of ceruloplasmin in cirrhosis and early-stage HCC serum samples (A–G). Ratio of fucosylated to non-fucosylated form of these glycopeptides (H, I). [file Image_3.tif]

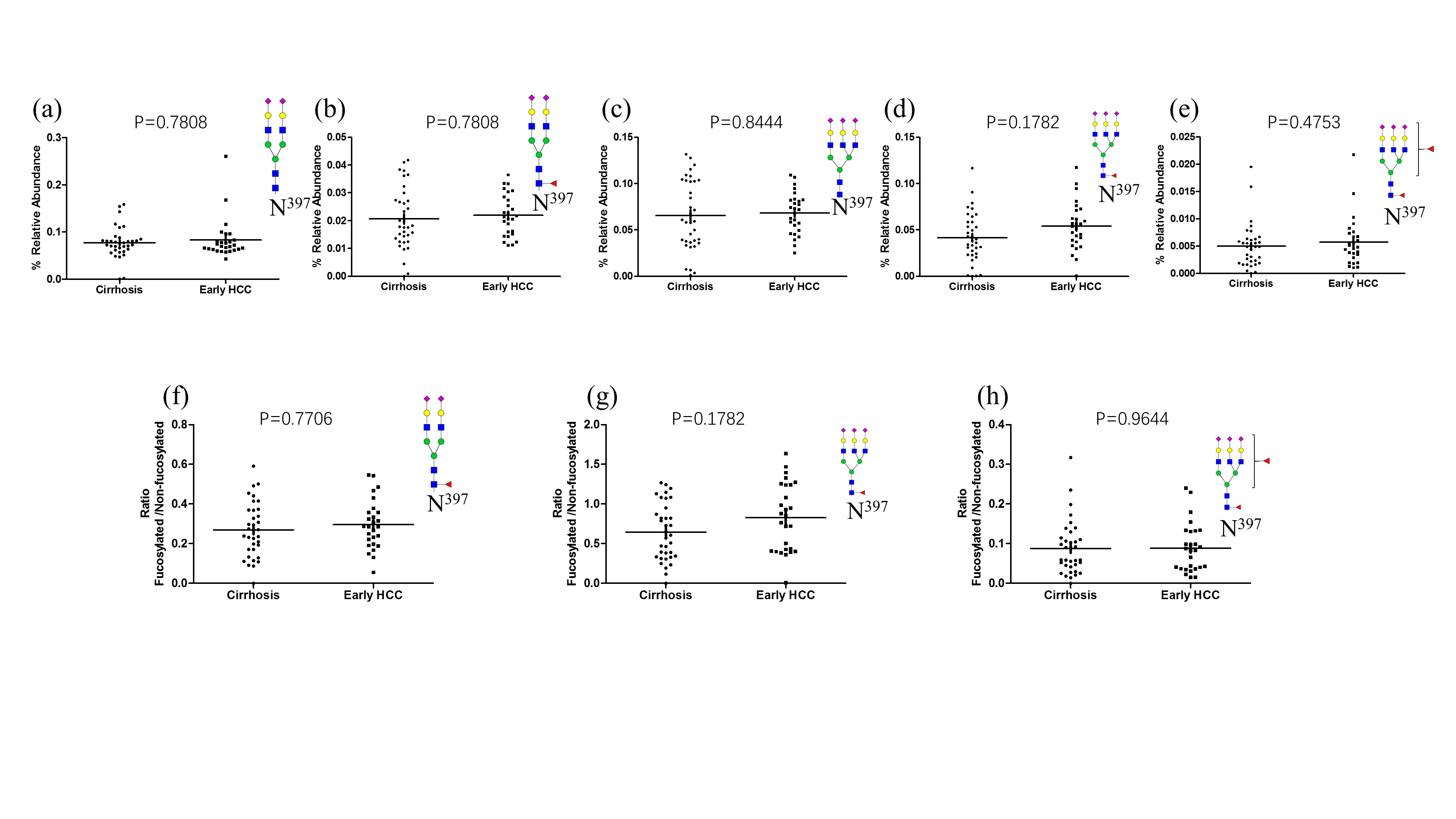

Supplement: Supplementary Figure S4 — Relative abundance of bi- and tri-antennary glycopeptides EN397LTAPGSDSAVFFEQGTTR of ceruloplasmin in cirrhosis and early-stage HCC serum samples (A–E). Ratio of fucosylated to non-fucosylated forms of these glycopeptides (F–H). [file Image_4.tif]

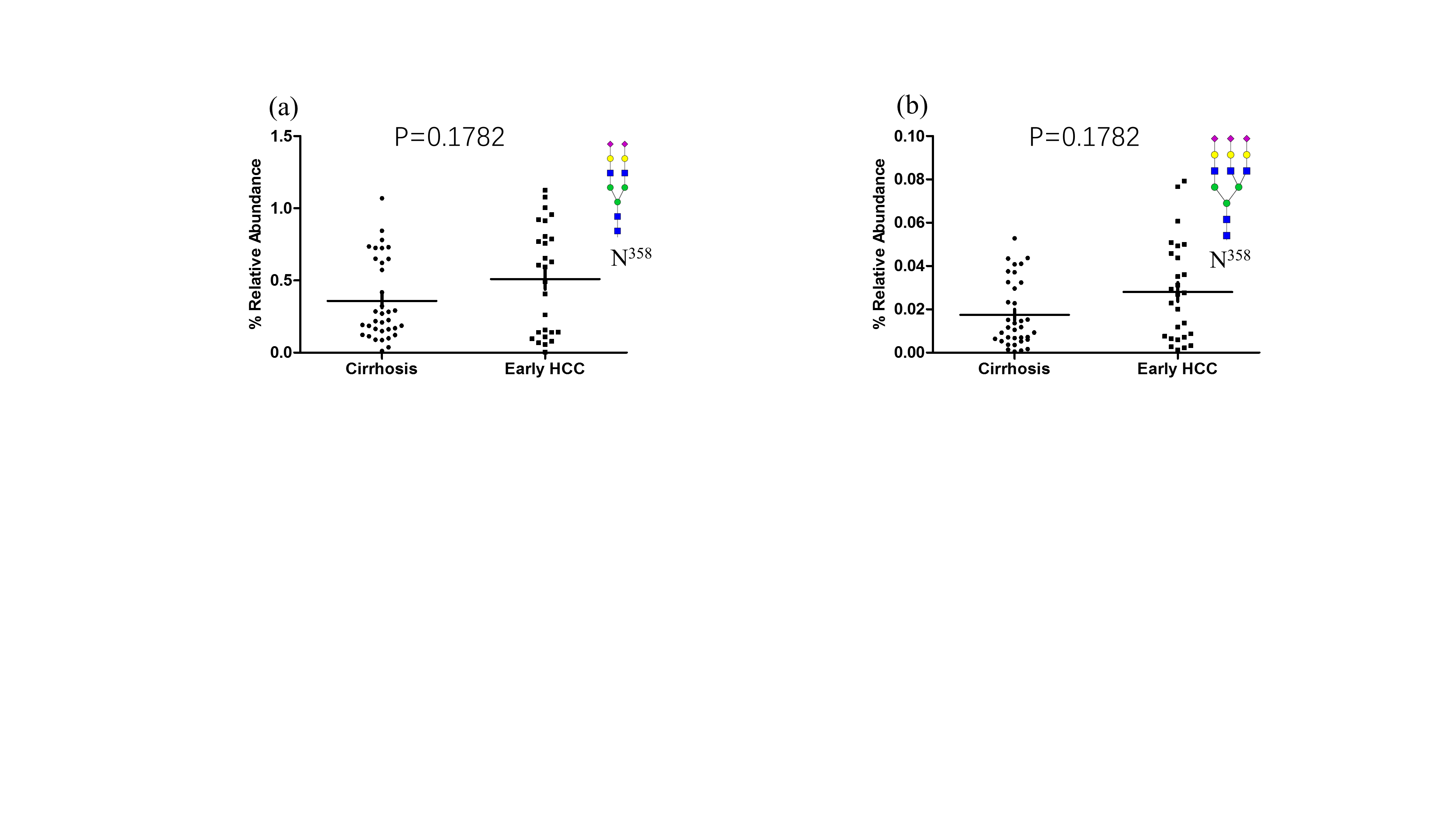

Supplement: Supplementary Figure S5 — Relative abundance of bi- and tri-antennary glycopeptides AGLQAFFQVQECN358K of ceruloplasmin in cirrhosis and early-stage HCC serum samples (A, B). [file Image_5.tif]
